# COP-22 Alleviates d-Galactose–Induced Brain Aging by Attenuating Oxidative Stress, Inflammation, and Apoptosis in Mice

**DOI:** 10.1007/s12035-024-03976-1

**Published:** 2024-02-12

**Authors:** Yazhong Ma, Xiaotong Wang, Xin Li, Xi Chen, Zhifeng Teng, Xuekun Wang, Jie Yang, Guoyun Liu

**Affiliations:** 1 School of Pharmaceutical Sciences, Liaocheng University, 1 Hunan Street, Liaocheng, 252059 Shandong China; 2 Liaocheng Key Laboratory of Quality Control and Pharmacodynamic Evaluation of Ganoderma Lucidum, Liaocheng University, 1 Hunan Street, Liaocheng, 252059 Shandong China

**Keywords:** Anti-aging, Brain aging, Oxidative stress, Inflammation, Apoptosis, COP-22, Curcumin derivative

## Abstract

Aging is a natural and inevitable process of organisms. With the intensification of population aging, research on aging has become a hot topic of global attention. The most obvious manifestation of human aging is the aging of brain function, which has been linked to the development of neurodegenerative diseases. In this study, COP-22, a mono-carbonyl curcumin derivative, was evaluated for its anti-aging ability, especially its ability to resist brain aging induced by D-galactose (D-gal) in mice. For brain protection, COP-22 could resist D-gal–induced oxidative stress by increasing the activity of antioxidative defense enzymes and enhancing antioxidant capacity in the brain tissue; COP-22 could improve the dysfunction of the cholinergic system by decreasing the increased activity of acetylcholinesterase and increasing the reduced content of acetylcholine induced by D-gal; and COP-22 could protect nerve cells of the brain. Further, western blot was used to determine related proteins of the brain. We found that COP-22 could effectively protect against brain injury (SIRT1, p53, p21, and p16) by inhibiting oxidative stress (Nrf2 and HO-1), inflammation (IL-6 and TNF-α), and apoptosis (Bax and caspase-3) in D-gal–induced aging mice. Additionally, COP-22 demonstrated the ability to reduce oxidative stress in serum and liver caused by D-gal, as well as relieve the damages in the liver and kidney induced by D-gal. These results indicated that COP-22 had potential anti-aging activity and could be used in the therapy of aging and aging-associated diseases like Alzheimer disease.

## Introduction

From a perspective, aging is a process that spontaneously and inevitably occurs in organisms over time. It is characterized by functional decline and structural degeneration [1] and decreased adaptability and resistance. From a pathological perspective, aging can be attributed to various factors such as infection and injury, stress and strain, weakened immune response, malnutrition, and metabolic disorders [2–6]. With the intensification of population aging, research on aging has become a hot topic of global attention. The most obvious manifestation of aging in the human body is the aging of brain function, which has been implicated in the progression of neurodegenerative diseases, including Alzheimer’s disease and Parkinson’s disease [7–11].

The D-galactose (D-gal)–induced aging mouse model has been the commonly used model for studying aging in recent years [12–14]. Accumulating evidence indicated that the aging changes induced by chronic administration of D-gal were similar to natural aging in animals [15, 16], and the brain aging processes induced by D-gal in mice are similar to these in humans [17, 18], including increased oxidative stress, neuronal degradation, inflammation, apoptosis, and so on [14, 17–19].

**Fig. 1:**
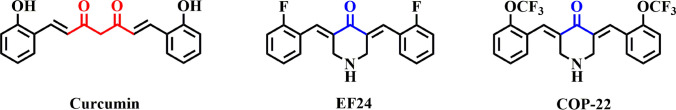
Chemical structures of curcumin and its derivatives

Curcumin is a kind of yellow polyphenolic compound extracted from *Curcuma longa* species with different biological properties, including anti-cancer, anti-inflammatory, antioxidant, anti-neurodegenerative, anti-aging activities, and so on [20, 21]. However, the poor bioavailability of curcumin limits its biomedical applications, due to the presence of β-diketone moiety in its chemical structure [22, 23]. Therefore, many mono-carbonyl curcumin derivatives have been designed, studied, and developed with improved biological activities in preventing and treating various diseases [24–27]. EF24 (Fig. 1), a potent synthetic curcumin derivative, is tenfold more anti-cancer potent than curcumin [28]; and was also a potent anti-aging agent [29]. COP-22 (Fig. 1) is also a synthetic curcumin derivative with low cytotoxicity and strong antioxidative and anti-inflammatory activity [30, 31]. Our previous works have demonstrated that COP-22 can protect Raw264.7 cells from inflammation induced by lipopolysaccharide (LPS) and oxidative stress induced by *tert*-butyl hydroperoxide and mice from ulcerative colitis induced by dextran sulfate sodium (DSS) [32] and acute liver injury induced by LPS/D-galactosamine.

In this research, we investigated the anti-aging activity of COP-22, in particular the protective effects of the brain, in the model of aging induced by D-gal in mouse. We evaluated the levels of the total antioxidant capacity (T-AOC), superoxide dismutase (SOD), catalase (CAT), malondialdehyde (MDA), and glutathione peroxidase (GPx), which are regarded as oxidative stress biomarkers of serum, liver, and brain. Moreover, we analyzed the histopathological changes of the brain stained with HE and Nissl. Furthermore, we evaluated the expressions of aging-, oxidative stress-, inflammation-, and apoptosis-associated markers in the brain tissue.

## Method

### Animal

C57bl/6j mice (male, 7-month-old, about 30 g) were purchased from Pengyue Experiment Animal Breeding Co. LTD. The mice were fed in the experimental animal house with a constant temperature of 23 ± 2 °C, and a 12-h dark/light cycle, and no restriction on access to a standard diet and water was provided. For the care and use of laboratory animals, all of the animal experiments were conducted in accordance with the guide of the national institute. The experimental protocols were backed by the ethics board of Liaocheng University.

### Treatment of D-gal–induced aging of mice

After the adopted feed for 1 week, C57bl/6j mice were divided randomly into five groups (*n* = 8). All of the mice but those raised in the group of control received daily intraperitoneal injection of 500 mg/kg D-gal (Amresco), and the mice raised in the group of 50, 100, or 200 mg/kg received daily oral administration of corresponding concentrations of COP-22 dissolved in physiological saline for 10 weeks. COP-22 was synthesized according to our previously published paper [30]. The mice were decapitated after the last administration. The blood was collected in a 1.5-mL centrifuge tube containing heparin. After storage for 2 h at room temperature, the blood was centrifuged at 4 °C at 1000* g* for 10 min, and the supernatant was collected as the serum for subsequent experiments. Half of the liver, kidney, and brain were collected in cryotubes and stored in liquid nitrogen. They were ground into 10% tissue homogenate supplemented with protease inhibitors (Beyotime) using a handheld homogenizer (Beyotime), and then centrifuged at 4 °C at 12,000* g* for 10 min. The tissue homogenate supernatant was used to detect biochemical parameters. Another half of them were collected in 5-mL centrifuge tubes containing 4% paraformaldehyde fix solution (Beyotime) for histopathological analysis.

### Histopathology Analysis

Brain, liver, and kidney were taken from decapitated mice, and then soaked in 4% paraformaldehyde fix solution, embedded in paraffin, and then sectioned. Stain the sections obtained from the brain, liver, and kidney with hematoxylin–eosin (H&E) and obtained from the brain with Nissl; then those sections stained were photographed by the Olympus microscope (BX53 + DP80).

### Measurement of Aspartate Aminotransferase (AST)/Alanine Aminotransferase (ALT)

According to the instruction (Applygen), add 24 μL of the standard solution into the standard well, 4 μL serum, and 20 μL buffer solution into the sample well, and 4 μL serum into the reference well. After incubation at 37 °C for 30 min, 20 μL of the chromogen solution was added into all of the wells, and 20 μL buffer solution was added into the reference well. After incubation at 37 °C for 20 min, 200 μL of the stop solution was added into all of the wells. Then, the mixture was incubated at room temperature for 10 min and measured at 520 nm by a microplate reader (BiotekSynergy H1).

### Measurement of BUN

According to the instruction (Nanjing Jiancheng Bioengineering Institute), 1 mL of reagent I and reagent II was added into 20 μL of the serum. Then, the mixture was heated at 100 °C for 15 min, lowered with cold water, and measured at 520 nm by a microplate reader (BiotekSynergy H1).

### Measurement of Creatinine (CRE)

According to the instruction (Nanjing Jiancheng Bioengineering Institute), 6 μL serum or standard was added into 180 μL reagent 1. The mixture was incubated at 37 °C for 5 min and measured at 546 nm (A1). Then, the mixture was added to 60 μL reagent II, incubated at 37 °C for 5 min, and the absorbance of the mixture was measured at 546 nm (A2). Δ*A* = A2 − 186*A1/246; the content of CRE (μmol/L) = Δ*A*_*sample*_/Δ*A*_*standard*_ * 442.

### GPx Assay

Adjust room temperature to 25 °C. Forty microliters of the detection buffer (Beyotime), 10 μL tissue homogenate supernatant, and 40 μL detection working solution of GPx were added into 96-well plates. Following the instruction of the reagent kit, the mixture solution was incubated for 15 min; add 10 μL 30 mM peroxide reagent, and continuously measure the absorbance at 340 nm for 5 min by a multiwall plate reader (Biotek Synergy H1). The activity of GPx in sample = (Δ*A*_340_/min)/(*ε*^μM^ × L(cm)). The *ε*^μM^ of NADPH at A340 nm is 0.00622 μM^−1^ cm^−1^, and the height of liquid in 96-well plates is 0.276 cm.

### T-AOC Assay

Detect the total antioxidant capacity in serum and tissue supernatant with the T-AOC detection kit (Beyotime). At first, following the instructions of the reagent kit, the working solution was prepared before 16 h. Add 10 μL serum or tissue homogenate supernatant to 200 μL working solution in 96-well plates. The mixture was incubated for 6 min and measured at 734 nm by a microplate reader (Biotek Synergy H1).

### T-SOD Assay

Following the instructions of the reagent kit (Beyotime), add NBT/enzyme working solution, sample, and reaction start working solution into 96-well plates, and the mixture was incubated at 37 °C for 30 min. Finally, the mixture was measured at 540 nm by a microplate reader (Biotek Synergy H1).

### MDA Assay

Add 0.1 mL tissue homogenate supernatant and 0.2 mL MDA working solution (Beyotime) in 1.5-mL centrifuge tubes, and then mix. The mixture was heated at 100 °C for 15 min, and lowered to room temperature using flowing water. After centrifuging for 10 min at 1000* g* at 4 °C, 200 μL supernatant was added into 96-well plates. Finally, the absorbance was measured at 532 nm by a microplate reader (Biotek Synergy H1).

### CAT Assay

Add 10 μL tissue homogenate supernatant and 30 μL CAT detection buffer (Beyotime) into 1.5-mL centrifuge tubes, and then 10 μL 250 mM hydrogen peroxide solution was added for 5 min at 25 °C. In order to terminate the reaction, 450 μL stop solution was added. Then, 10 μL of the terminated reaction solution was pipetted to another centrifuge tube with 40 μL CAT detection buffer. Pipette 10 μL from the 50-μL mixture system in the previous step and 200 μL chromophoric reagent to 96-well plates, and then the mixture was incubated at 25 °C for 15 min and measured at 520 nm by a microplate reader (Biotek Synergy H1).

### Acetylcholine (Ach) Assay

According to the instructions provided by the manufacturer (Nanjing Jiancheng Bioengineering Institute), the supernatant of brain tissue homogenate was prepared in the ratio of brain tissue (mg) to reagent VI (mL) = 1:9 (w/v), and centrifuged at 3000 rpm for 10 min. The supernatant was taken for subsequent detection. The detection wells were divided into blank wells, standard wells, sample wells, and reference wells. First, 50 μL water was added into the blank well; 40 μL water and 10 μL standard solution were added into the standard well, and 25 μL tissue supernatant was added into the sample well and control well. Then, 100 μL working solution was added into all wells except the reference well. After mixing and incubation at room temperature for 15 min, 50 μL of the termination solution and chromogenic solution was added into all wells, and 25 μL clarification solution was added into the sample wells and control wells, and then replenish 100 μL the reagent working solution to the reference well. Measure the absorbance at 550 nm after mixing and incubation for 10 min at room temperature.

$$Ach\left(\mu g/mgprot\right)=\left({A}_{determination}-{A}_{reference}\right)/\left({A}_{standard}-{A}_{blank}\right)\times {C}_{standard}/{C}_{the protein concentration of the sample}$$

### Acetylcholinesterase (AchE) Assay

Take the prepared 10% brain tissue homogenate supernatant for the detection of AchE enzyme activity, and the instruction provided by the reagent kit was strictly followed during all experimental operations (Nanjing Jiancheng Bioengineering Institute). The detection wells were divided into blank wells, standard wells, sample wells, and reference wells. Except for the reference well, 10 μL 10% brain tissue homogenate supernatant, 50 μL substrate buffer, and 50 μL chromogenic solution were added into each well. After incubating at 37 °C for 6 min, 3 μL inhibitor and 10 μL transparent agent were added into all detection wells, and 10 μL 10% brain tissue homogenate supernatant was added into the control well. The absorbance at 412 nm was measured after mixing and incubating at room temperature for 15 min.

$$AchE (U/mgprot) = ({A}_{determination}-{A}_{control})/({A}_{standard}-{A}_{blank}) *{C}_{standard}/{C}_{the protein concentration of the sample}$$

### Western Blotting Assay

The tissues of sacrificed mice were lysed by radio immunoprecipitation assay (RIPA) buffer (Beyotime), and the protein extracted from the tissues was examined by western blot assay. Primary antibodies are as follows: SIRT1 (Wanleibio, WL00599, 1:1000), p53 (Wanleibio, WL01919, 1:1000), p21 (Wanleibio, WL0362, 1:1000), p16 (Wanleibio, WL01418, 1:1000), HO-1 (Wanleibio, WL02400, 1:1000), Nrf2 (Beyotime, AF7623, 1:1000), Caspase-3 (Wanleibio, WL02117, 1:1000), Bax (Cell Signaling Technology, 2772S, 1:1000), interleukin (IL)-6 (Wanleibio, WL02841, 1:1000), tumor necrosis factor-α (TNF-α) (Wanleibio, WL01581, 1:1000), β-actin (Servicebio, GB15003-100, 1:1000).

### Statistical Analysis

The data were expressed as the mean ± SD of at least three independent experiments. Statistical significances were analyzed using one-way ANOVA followed by Dunnett’s multiple comparison test. *p* < 0.05 was considered statistically significant.

## Results

### Effects of COP-22 on Liver and Kidney Functions of Aging Induced by D-gal in Mice

#### Effects of COP-22 on AST, ALT, BUN, and CRE Levels in the Serum

**Fig. 2:**
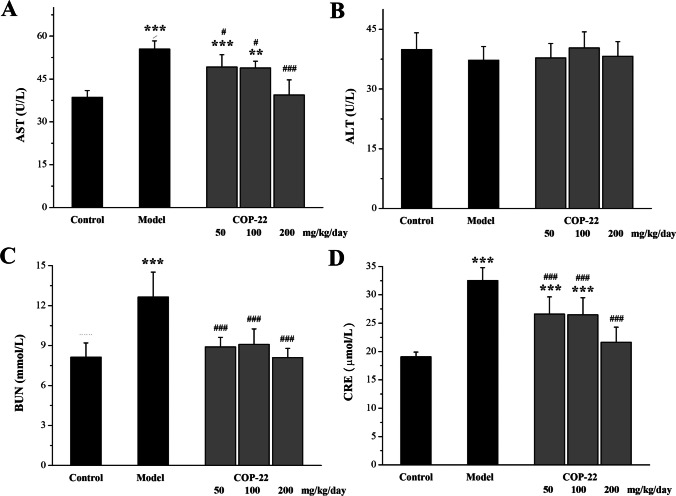
The expressions of ALT (**A**) and AST (**B**) in serum for the liver fuction, and BUN (**C**) and CRE (**D**) in serum for the kidney function after administration of COP-22 and D-gal. *p < 0.05; **p < 0.01; ***p < 0.001, compared with the control group. #*p* < 0.05; ^##^*p* < 0.01; ^###^*p* < 0.001, compared with the model group

To regard kidney and liver functions, we examined the biochemical parameters (AST, ALT, BUN, and CRE levels) in the serum. As shown in Fig. 2A, for the liver damage, the AST level was increased in the model group due to chronic administration by D-gal. After the D-gal–induced mice were treated with COP-22, the increase of AST level was reduced, and this was concentration dependent. However, there was no statistical significance (*p* > 0.05) in the expression of ALT among groups (Fig. 2B). As shown in Fig. 2C and D, for the kidney damage, the BUN and CRE levels induced by D-gal were increased. After the D-gal–induced mice were treated with COP-22, the increases of BUN and CRE levels were reduced.

#### Effects of COP-22 on Liver and Kidney Histopathological Alterations

**Fig. 3:**
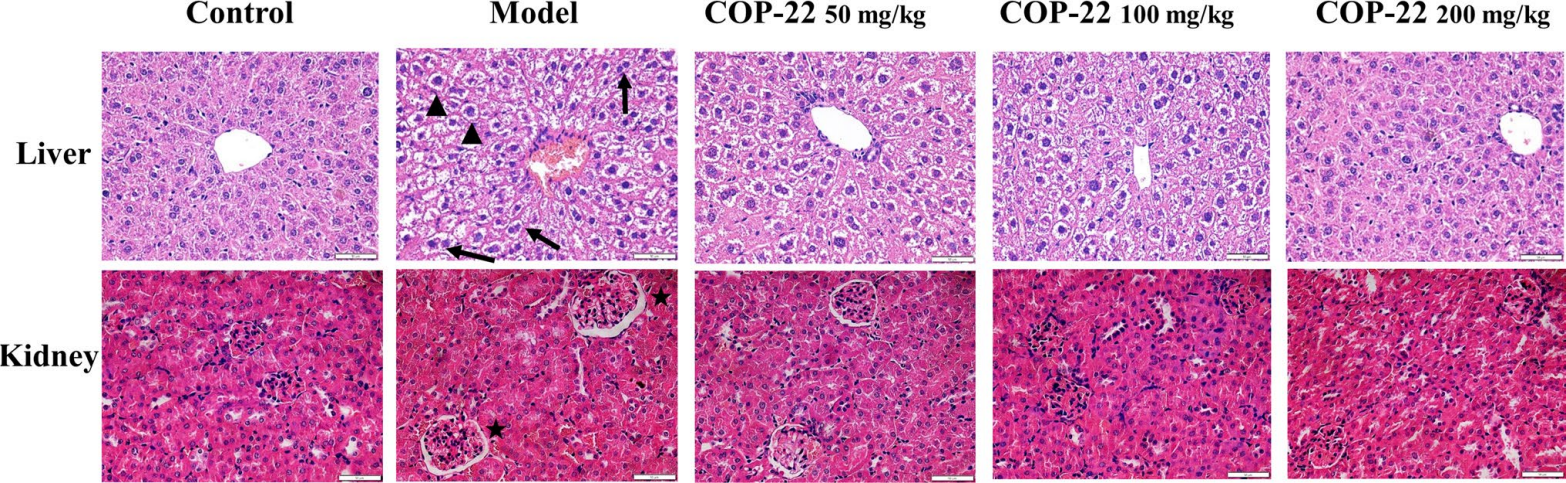
Effects of COP-22 treatment on pathological changes in the liver and kidney. HE staining (triangle, swollen; arrow, binuclear phenomenon; star, an increase in glomerular volume) (Scale bar: liver, 50 μm; kidney, 50 μm)

HE staining of the liver and kidney was used for histopathological study. As illustrated in Fig. 3, there were no abnormalities in the morphology of control liver tissue, but the cells in the model group are swollen (triangle) and have a binuclear phenomenon (arrow). The morphology of the kidney tissue from the control group was normal, but the morphology of the kidneys of model group showed an increase in glomerular volume (star). However, COP-22 treatment can significantly improve these pathological conditions.

### Effects of COP-22 on Oxidative Stress Biomarkers in the Serum and Livers of Aging Induced by D-gal in Mice

**Fig. 4:**
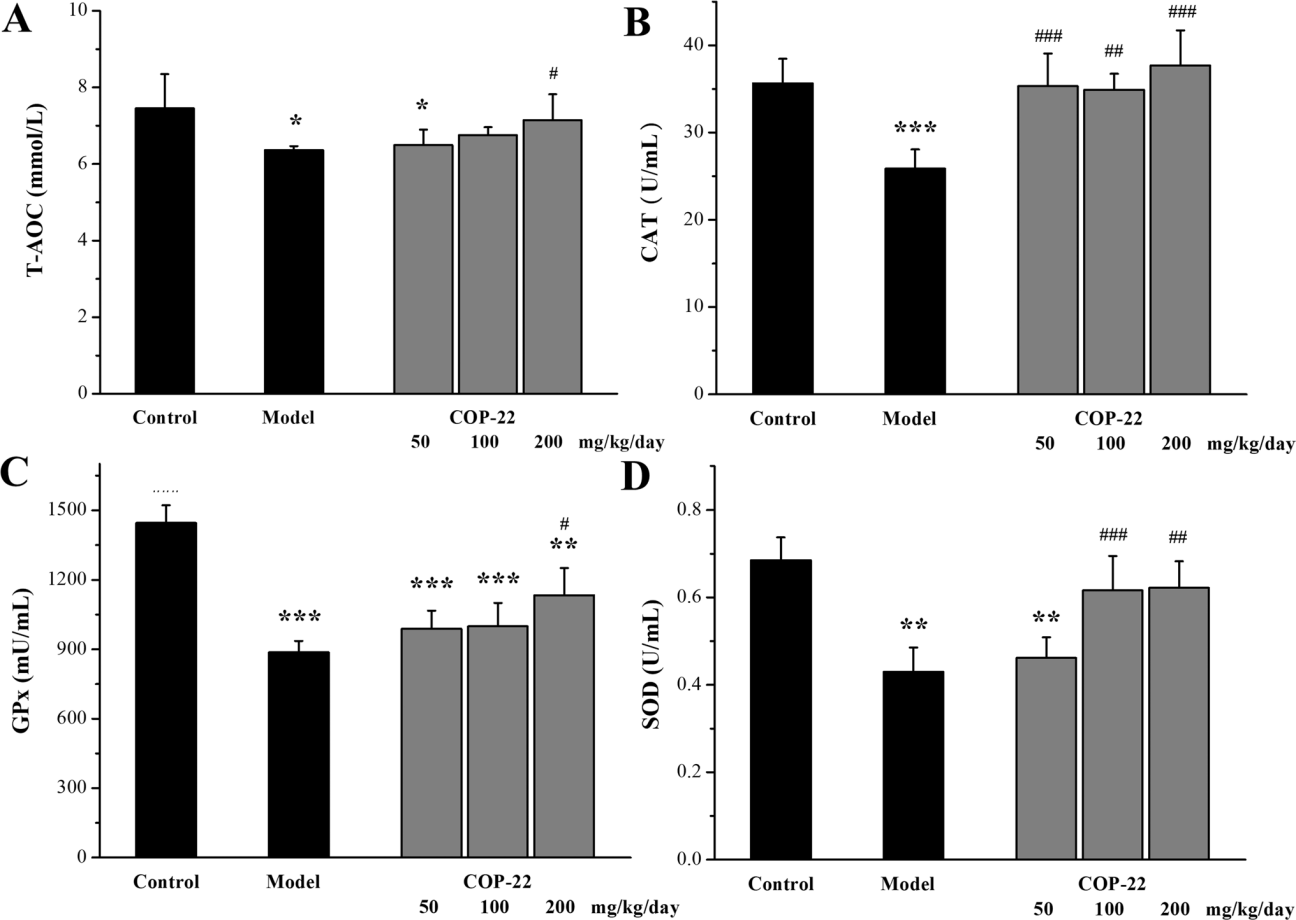
Effects of COP-22 on T-AOC (**A**), CAT (**B**), GPx (**C**), and SOD (**D**) levels in the serum of D-gal–induced aging mice. *p < 0.05; **p < 0.01; ***p < 0.001, compared with the control group. ^#^*p* < 0.05; ^##^*p* < 0.01; ^###^*p* < 0.001, compared with the model group

As illustrated in Fig. 4, the reduction of T-AOC, GPx, CAT, and SOD in the serum of aging mice was induced by D-gal. Treatment with COP-22 reversed these changes in oxidative stress biomarkers in a dose-dependent manner.

**Fig. 5:**
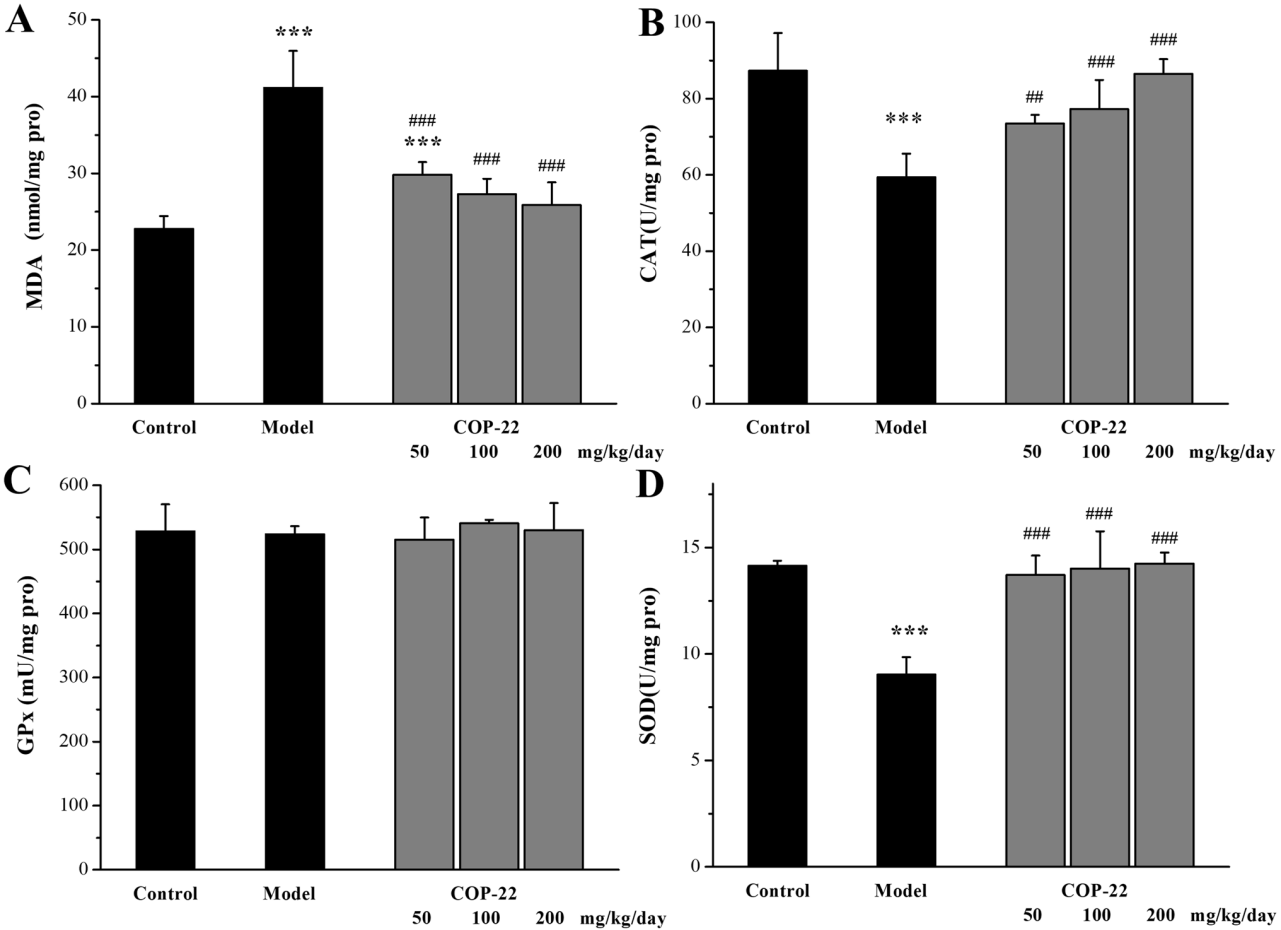
Effects of COP-22 on MDA (**A**), CAT (**B**), GPx (**C**), and SOD (**D**) levels in the liver tissue of D-gal–induced aging mice. *p < 0.05; **p < 0.01; ***p < 0.001, compared with the control group. ^#^*p* < 0.05; ^##^*p* < 0.01; ^###^*p* < 0.001, compared with the model group

As illustrated in Fig. 5A, the increase of MDA in the liver of aging mice was induced by D-gal. Treatment with COP-22 reduced the increase of MDA in a dose-dependent manner. As illustrated in Fig. 5B and D, the reduction of CAT and SOD in the liver of aging mice was induced by D-gal. Treatment with COP-22 reversed these reductions in a dose-dependent manner. There was no statistical significance (*p* > 0.05) in the levels of GPx among groups (Fig. 5C).

### Effects of COP-22 on Protective Brain of D-gal–Induced Aging in Mice

#### Effects of COP-22 on Oxidative Stress Biomarkers in the Brain

**Fig. 6:**
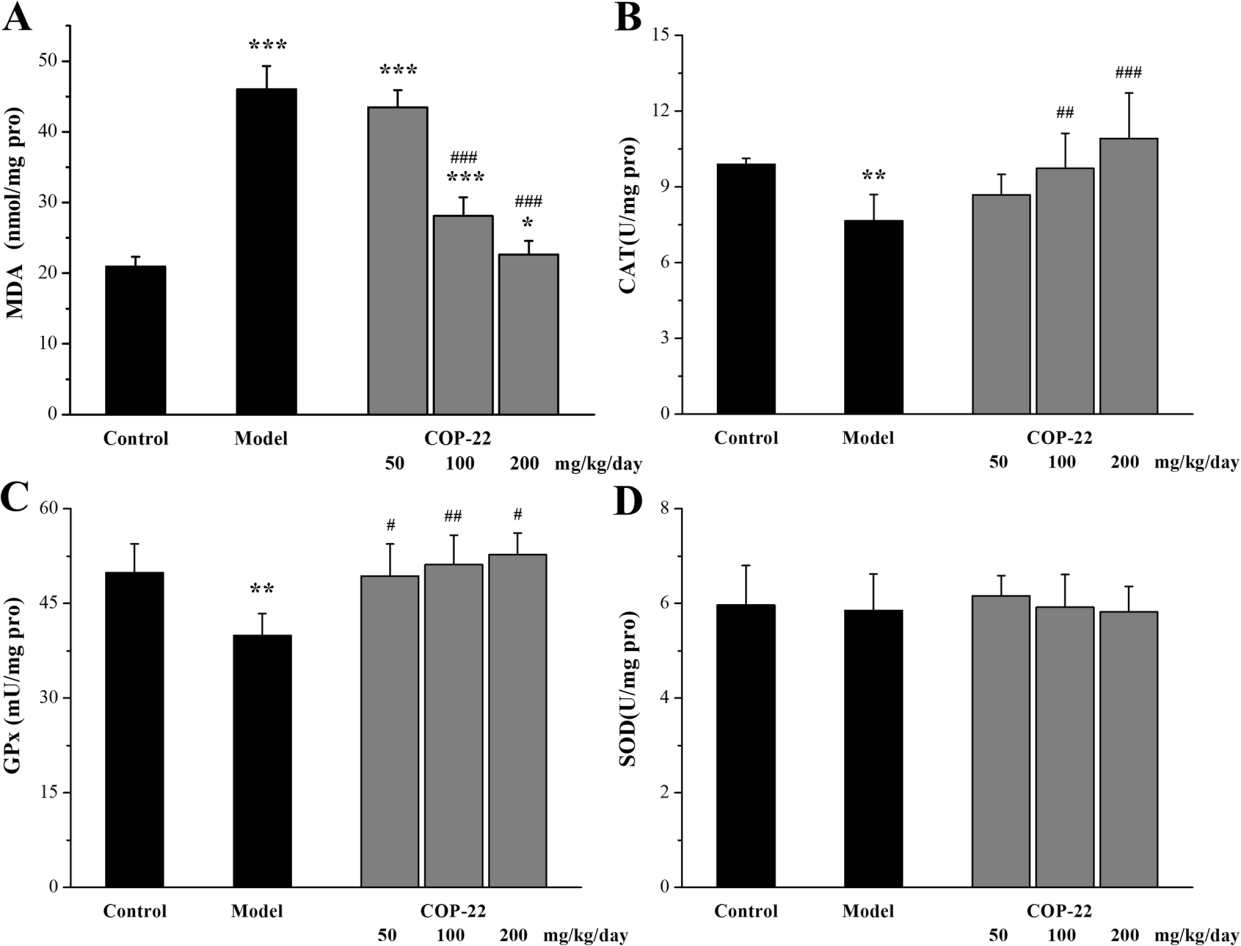
Effects of COP-22 on MDA (**A**), CAT (**B**), GPx (**C**), and SOD (**D**) levels in the brain tissue of D-gal–induced aging mice. *p < 0.05; **p < 0.01; ***p < 0.001, compared with the control group. ^#^*p* < 0.05; ^##^*p* < 0.01, ^###^*p* < 0.001, compared with the model group

As illustrated in Fig. 6, COP-22 reduced the increase of MDA induced by D-gal, and increased the reduction of CAT and GPx induced by D-gal in aging mice, in a dose-dependent manner. There was no statistical significance (*p* > 0.05) in the levels of SOD among groups (Fig. 6D).

#### Effects of COP-22 on the Expression of Ach Level and the Activity of AchE in the Brain

**Fig. 7:**
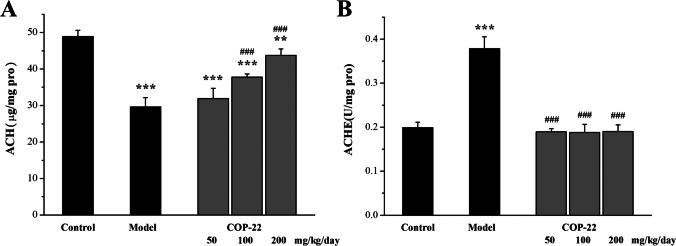
Effects of COP-22 on Ach (**A**) and AchE (**B**) levels in the brain tissue. *p < 0.05; **p < 0.01; ***p < 0.001, compared with the control group. ^#^*p* < 0.05; ^##^*p* < 0.01; ^###^*p* < 0.001, compared with the model group

As illustrated in Fig. 7, a decrease in Ach level and an increase in AchE activity in the brain were caused by D-gal. COP-22 increased the decrease of Ach in a dose-dependent manner. Only 50 mg/kg/day COP-22 reversed the increase of the activity of AchE.

#### Effects of COP-22 on Histopathological Alternations in the Brain

**Fig. 8:**
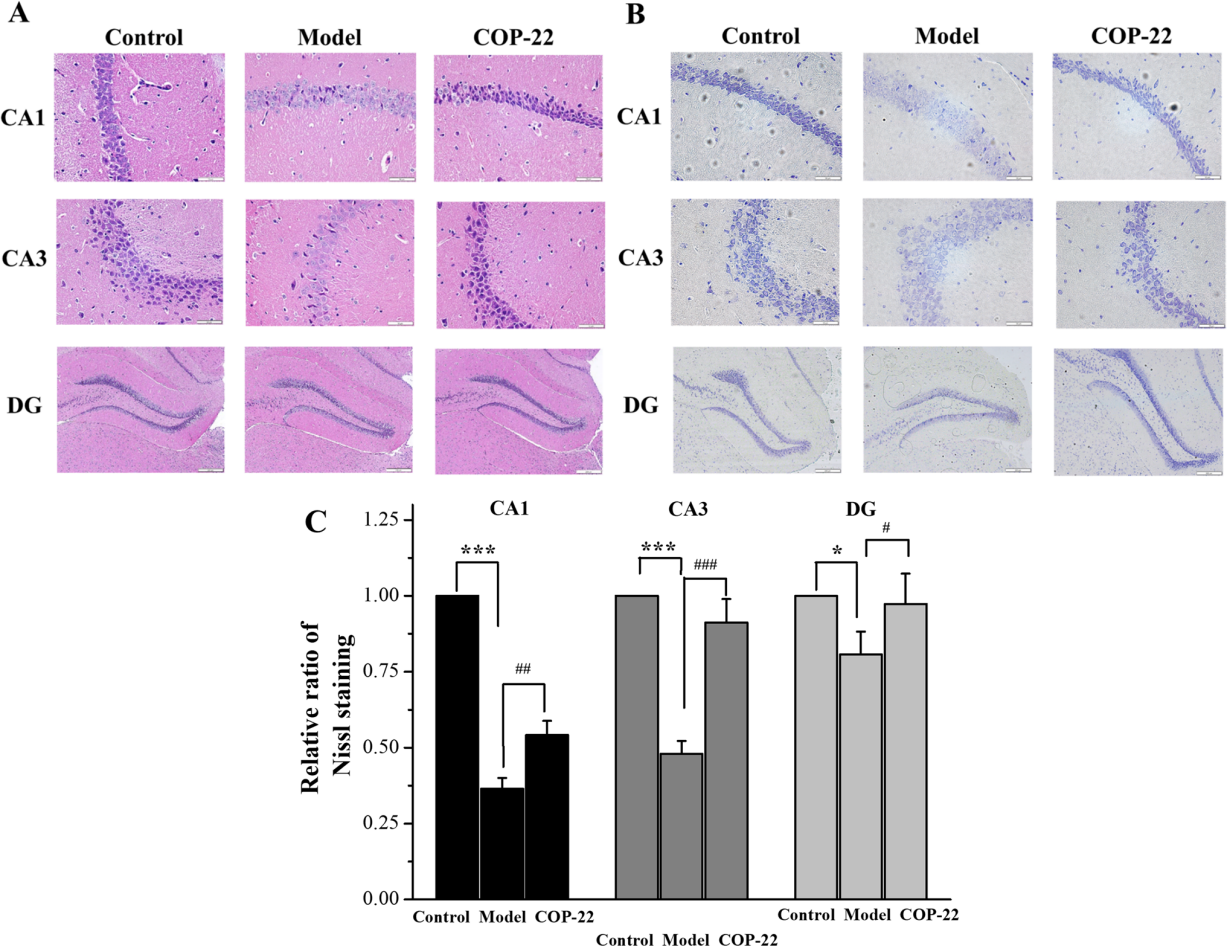
Effects of COP-22 treatment on pathological changes in the CA1, CA3, and DG areas of the hippocampus. **A** HE staining. **B** Nissl staining (scale bar: CA1, 50 μm; CA2, 50 μm; DG, 200 μm). **C** Relative ratio of Nissl staining of CA1, CA3, and DG areas. *p < 0.05; **p < 0.01; ***p < 0.001, compared with the control group. ^#^*p* < 0.05; ^##^*p* < 0.01;^###^*p* < 0.001, compared with the model group

As illustrated in Fig. 8A, the morphology of CA1, CA3, and DG areas in the hippocampus of the control group was normal. However, the model group showed that the structure of the hippocampus was loose, the membrane boundary was faint, and the morphology of the DG area changed, which demonstrated that D-gal treatment caused damages to the hippocampal neurons. Treatment with 200 mg/kg/day COP-22 significantly improved these pathological conditions.

As shown in Fig. 8B and C, the model group was only slightly stained by Nissl, but the 200 mg/kg/day COP-22 treatment groups could improve this situation.

#### Effects of COP-22 on Aging, Oxidative Stress, Inflammation, and Apoptosis-Related Proteins

**Fig. 9:**
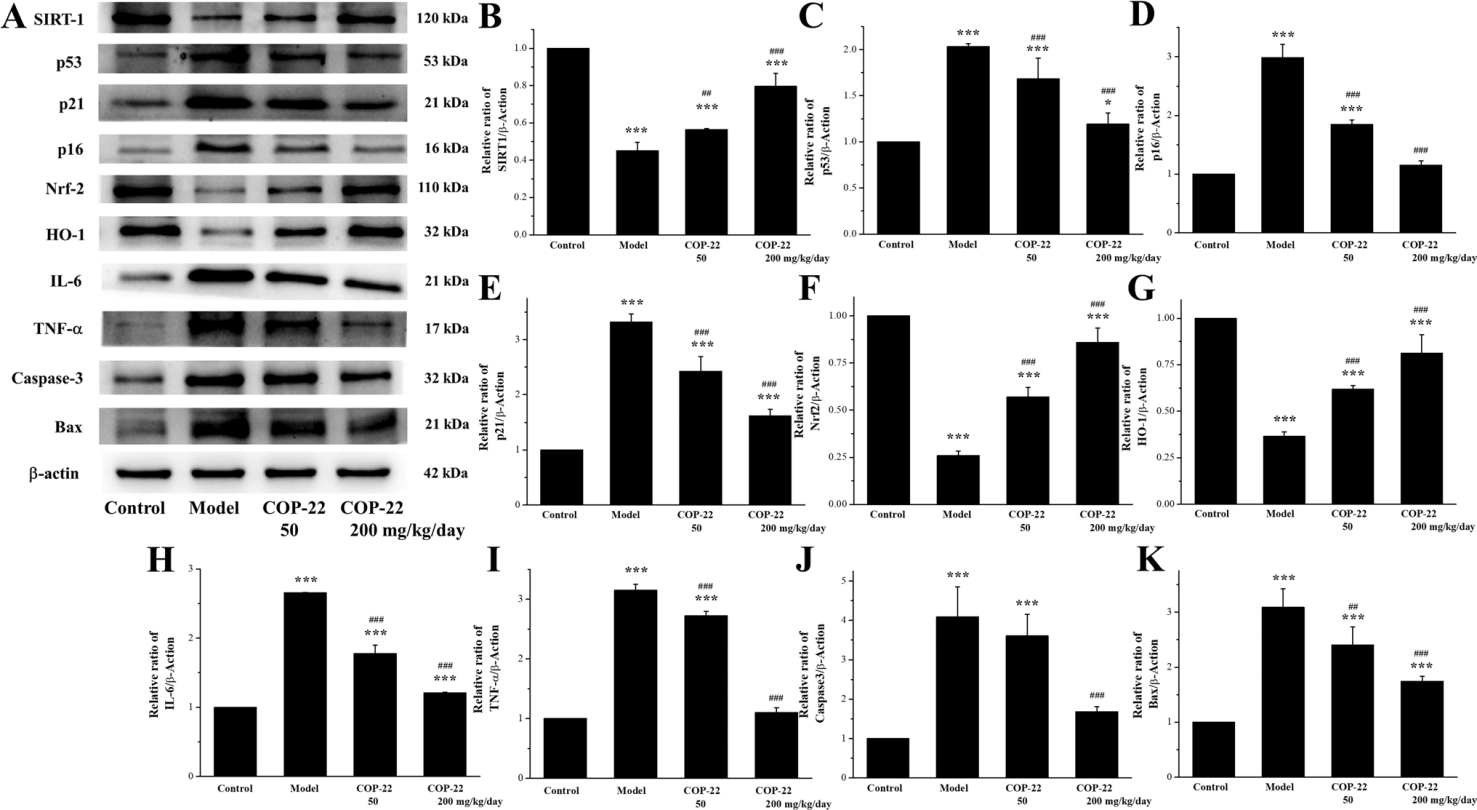
Effects of COP-22 on aging (SIRT1, p53, p21, and p16), oxidative stress (Nrf2 and HO-1), inflammation (IL-6 and TNF-α), and apoptosis (caspase 3 and Bax) in the brain tissue. *p < 0.05; **p < 0.01; ***p < 0.001, compared with the control group. ^#^*p* < 0.05; ^##^*p* < 0.01; ^###^*p* < 0.001, compared with the model group

As shown in Fig. 9A–E, we analyzed the senescence-associated proteins SIRT1, p16, p21, and p53 in the brain tissue using western blot assay. In D-gal–induced aging mice, the level of SIRT1 was decreased, and the level of p16, p21, and p53 was increased. The treatment of COP-22 effectively reversed the reduced expression of SIRT1 and the upregulation of expressions of p16, p21, and p53 in a dose-dependent manner.

As shown in Fig. 9A, F, and G, the oxidative stress–associated proteins HO-1 and Nrf2 in the brain were analyzed by western blot assay. The reductions of the expression levels of Nrf2 and HO-1 were induced by D-gal, while COP-22 reversed these changes.

As shown in Fig. 9A, H, and I, the inflammation-associated proteins TNF-α and IL-6 in the brain tissue were analyzed by western blot assay. The increases of the expression levels of IL-6 and TNF-α were induced by D-gal, while COP-22 reversed these changes.

As shown in Fig. 9A, J, and K, the apoptosis-associated proteins caspase-3 and Bax in the brain tissue were analyzed by western blot assay. The increase of the expression levels of Bax and caspase-3 was induced by D-gal, while COP-22 reversed these changes.

## Discussion

In this study, we investigated the anti-aging activity of COP-22 in the D-gal–induced aging mouse model, especially its protective effect on brain aging. Injecting D-gal can lead to excessive production of ROS in mice, causing an imbalance in the redox system, leading to oxidative stress and accelerated aging, especially brain aging; this is because the brain is particularly susceptible to oxidative damage and a relative lack of antioxidant defense mechanisms.

Aging and age-associated diseases are primarily caused by oxidative stress [33]. The biomarkers of oxidative stress, T-AOC, an end product of lipid peroxidation (MDA), and the activities of antioxidative defense enzymes (GPx, SOD, and CAT) in the serum, liver, and brain of aging mice induced by D-gal were determined. In the model group, D-gal induced the reduction of T-AOC, CAT, GPx, and SOD and the increase of MDA. COP-22 could effectively reverse these changes, thereby protecting mice against D-gal–caused oxidative stress.

In addition to oxidative stress, inflammation also plays an important role in the aging process [34, 35], which can be explained by the imbalance between anti-inflammatory and pro-inflammatory factors [36]. The relationship between aging, oxidative stress, and inflammation is complex and interrelated. The brain aging is more sensitive, complex, and related with inflammation, oxidative stress, apoptosis, and so on [14, 17, 37, 38]. Furthermore, we further measured the expression of aging, oxidative stress, inflammation, and apoptosis proteins.

SIRT1, a protein related to mammalian aging, was downregulated by D-gal in the aging mice. According to reports, the SIRT1 deficiency can promote the expression of aging-related genes [39]. p53/p21 and p16/Rb pathways play the critical role in the aging process through cell cycle arrest [40, 41]. In the aging mice, the expressions of p16, p21, and p53 were upregulated, indicating cell cycle arrest and aging. COP-22 could upregulate the expression of SIRT1 and downregulate expressions of p16, p21, and p53. COP-22 could intervene in brain aging caused by D-gal and reduce the degree of aging.

Nrf2 is a key oxidative stress signaling pathway, and HO-1 is an important downstream phase II antioxidant enzyme. When the occurrence of oxidative stress is caused by D-gal in aging mice, the expressions of Nrf2 and HO-1 were downregulated. Therefore, these results indicated that COP-22 could protect the brain from D-gal–induced oxidative stress and aging by regulating the Nrf2 signaling pathway.

IL-6 and TNF-α are two kinds of cytokines, which have multiple functions such as regulating innate and adaptive immunity and participating in inflammatory reactions. In the aging mice, the expressions of IL-6 and TNF-α were upregulated; however, COP-22 could downregulate these expressions, which meant COP-22 effectively suppresses D-gal–induced inflammation to resist brain aging.

Bax, a pro-apoptotic type protein of the Bcl-2 family, was one of the most crucial regulatory genes in the process of regulating apoptosis. Caspase-3, in the process of apoptosis, was the most important apoptosis executing protease. D-gal caused the increases in expressions of Bax and Bcl-2 leading to brain apoptosis. COP-22 could downregulate the expressions of Bax and Bcl-2 and effectively resist D-gal–induced apoptosis to protect the brain. As mentioned above, COP-22 could delay brain aging by suppressing apoptosis, inflammation, and oxidative stress caused by D-gal in the aging mice.

Ach is a neurotransmitter, and it can be rapidly destroyed by cholinesterase. There is a large amount of Ach in human brain tissue; however, the content of Ach will decrease with the increase of age. The decrease in Ach is one of the reasons for brain function decline [42]. AchE, a crucial enzyme in biological nerve conduction, can degrade Ach between cholinergic synapses, block the excitatory effect induced by neurotransmitters on the postsynaptic membrane, and ensure the nerve signals can be transmitted normally within the organism [43, 44]. The changes of the Ach content and AchE activity in the brain have become one of the main indicators for studying human brain functional aging. In aging mice, D-gal reduced the content of Ach and upregulated the expression of AchE; however, COP-22 reversed these changes. COP-22 improved the dysfunction of the cholinergic system and protected the brain against aging.

In addition, histopathological alternations of the brain were analyzed by the staining of HE and Nissl. D-gal can decrease the surviving neurons of the hippocampus in previous studies [45]. Nissl bodies, large basophilic masses and granules within the cell body or dendrites of neurons, can serve as markers of neuronal functional status. Using the Nissl staining method to stain brain tissue can be used to evaluate the state of neurons [46]. The histopathological alternations in the brain stained by HE and Nissl were improved, indicating that COP-22 could resist the hippocampal neuron damages caused by D-gal, and protect nerve cells of the brain against D-gal–induced brain aging.

According to reports, injecting D-gal can cause damage to the liver and kidney [47, 48]. The biochemical parameters AST, ALT, BUN, and CRE in the serum and histopathological alterations of the liver and kidney were determined. COP-22 reduced the increases of AST, BUN, and CRE caused by D-gal in aging mice. And COP-22 improves those histopathological alterations, such as swollen and binuclear of liver cells and the increase in glomerular volume of the kidney. These results of biochemical parameters in the serum and histopathological analyses suggested that COP-22 could effectively reverse the liver and kidney damages in aging mice induced by D-gal.

## Conclusion

**Fig. 10:**
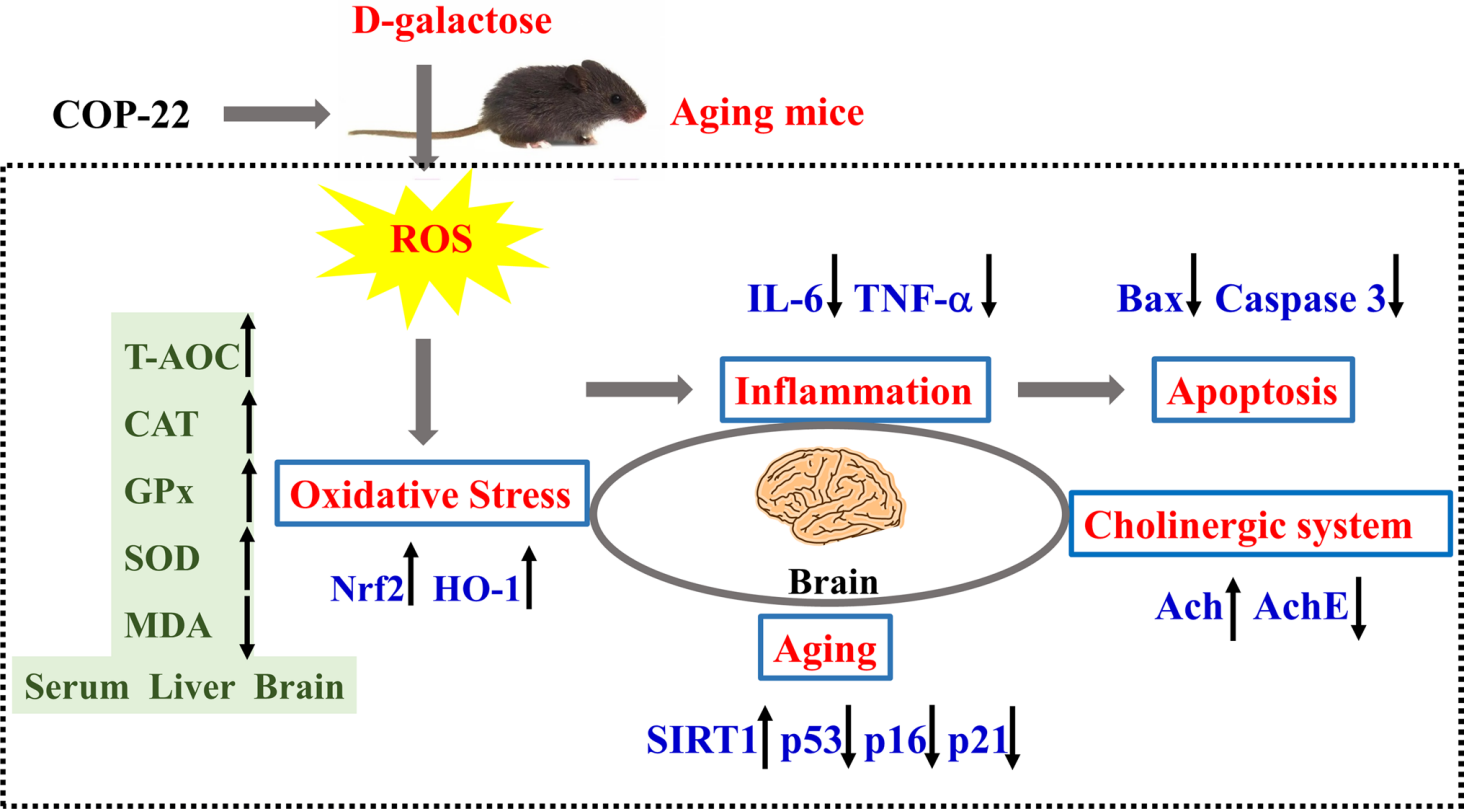
Possible mechanism underlying the protective effects of COP-22 intervention on D-gal–induced aging in mice

In this study, COP-22, a mono-carbonyl curcumin derivative, was evaluated for its anti-aging ability, especially its ability to resist brain aging, in the aging induced by D-gal in mice. The result suggested that COP-22 could reduce liver and kidney damages induced by D-gal. And COP-22 could resist oxidative stress induced by D-gal in serum and liver by increasing the activity of antioxidative defense enzymes and enhancing antioxidant capacity. For brain protection, COP-22 could also resist oxidative stress induced by D-gal in the brain tissue; COP-22 could improve the dysfunction of the cholinergic system by decreasing the increased activity of AchE and increasing the reduced content of Ach induced by D-gal, and COP-22 could protect nerve cells of the brain. Further, the western blot was used to determine related proteins of the brain. We found that COP-22 could effectively protect against brain injury (SIRT1, p53, p21, and p16) by inhibiting oxidative stress (HO-1 and Nrf2), apoptosis (caspase-3 and Bax), and inflammation (TNF-α and IL-6) in D-gal–induced aging in mice (Fig. 10).

To sum up, these results indicated that COP-22 had potential anti-aging activity, especially alleviating brain aging, via attenuating oxidative stress, inflammation, and apoptosis, which could be used in the therapy of aging and aging-associated diseases like Alzheimer disease. However, additional anti-aging molecular mechanisms and COP-22-loaded nanoparticles, with the improved dissolution and bioavailability, need to be explored in future investigations.

## Data Availability

Data will be made available under the reasonable request.
